# Determining spatial disparities and similarities regarding heat exposure, green provision, and social structure of urban areas - A study on the city district level in the Ruhr area, Germany

**DOI:** 10.1016/j.heliyon.2023.e16185

**Published:** 2023-05-19

**Authors:** Florian Klopfer, Antonia Pfeiffer

**Affiliations:** Department of Spatial Planning, TU Dortmund University, Dortmund, Germany

**Keywords:** Urban heat island (UHI), NDVI, Social vulnerability, Cluster analysis, Multiple burdens, Climate (in-)justice, Environmental (in-)justice

## Abstract

Heat islands and ongoing urbanization make cities places where the negative impacts of global climate change on society are becoming increasingly evident. Especially the interplay and potential multiplication of heat, low green provision, and the presence of socially deprived urban dwellers constitutes complex challenges. Emerging climate injustices and potential health issues require a powerful counter-reaction in form of adaptation action. For our study, we consider eight cities located in the densely populated and historically highly segregated Ruhr area in Western Germany, which is one of the largest metropolitan areas in Europe with a heterogeneous distribution of socio-spatial problems, economic potential, heat stress, and green infrastructures. We use land surface temperature (LST), data on green provision (normalized difference vegetation index (NDVI)), and social indicators to reveal the relationships between these indicators on the city district level (n = 275). Therefore, we first analyze the data regarding spatial autocorrelation (Moran's *I*) and clustering (Gi*) before calculating study area wide and city specific correlations between the three factors regarded. Finally, we conduct a cluster analysis (k-means) to disclose similar areas with or without multiple burdens. Our results show distinct disparities in heat exposure, green availability, and social status between city districts of the study area. We find strong negative correlations between LST and NDVI as well as between NDVI and social status. The relationship between LST and our social indicator remains ambiguous, affirming the necessity of further detailed studies. The cluster analysis furthermore allows for the visualization and classification of districts featuring similar characteristics regarding the researched components. We can discern in parts pronounced climate injustice in the studied cities, with a majority of people living in unfavorable environmental and socio-economic conditions. Our analysis supports governments and those responsible for urban planning in addressing climate injustice in the future.

## 1 Introduction

The interplay of ongoing climatic changes and urbanization creates a variety of challenges for urban areas around the globe. In its latest report the IPCC (Intergovernmental Panel on Climate Change) stresses that limiting global warming to 1.5°C above pre-industrial times until the end of the century is still possible, however, it also points out that the global surface temperature will nonetheless continue to rise at least until the 2050s [1]. At the same time, the UN estimates the global urban population share to be 56.2% in 2020 and projects it at 68.4% in 2050 [2]. During the period from 2015 to 2020, urban populations grew by almost 400 million people. Over 90% of this growth took place in less developed regions [3]. Urbanization is considered to induce vulnerability and exposure and in combination with climate change hazards is driving urban risk and impacts. As most rapid population growth is in areas where adaptive capacity is low, the most economically and socially marginalized are most affected by adverse climate change impacts [3]. Not only regarding climate change and urban heat, studies have shown the connection between environmental (multiple) stresses and the respective social situation of urban dwellers [[4], [5], [6], [7], [8]]. The socio-spatial concentration of such environmental burdens (e.g., heat, noise, air pollutants, lack of green spaces, poor housing conditions) corresponds with socially disadvantaged urban neighborhoods. Characteristic is both the increased level of pathogenic (e.g., air pollutants) and the lack of salutogenic (e.g., green spaces) environmental factors in such areas, which further increase the social vulnerability of residents and affects their general health [4]. To counteract increasing heat stress in cities, a fundamental intervention option is the utilization of the thermally dampening potential of green and water areas [9]. Predominantly low-sealed green areas provide important services for the local microclimate. Living in areas which are cooler and feature a higher vegetation cover is also associated with a reduced risk for heat related morbidity and mortality [10]. Adverse climate effects are not limited to generally rather deprived world regions. In the US, already more than ten years ago, heat was the number one natural hazard causing deaths [11]. In Germany, in the summer of 2003, approx. 9600 people died from heat-related issues [12] and approx. 8700 in 2018 [13].

Knowledge about the spatial patterns of heat hazards in form of urban heat islands (UHI), urban heat drivers or inhibiters like green spaces, and urbanites exposed to heat is crucial when it comes to addressing these issues from the planning side. Interventions for adaptation are necessary to meliorate the livability of urban spaces [14,15]. Regarding the characteristics of people potentially at risk, it is important to determine the existence of climate injustice in cities. The objective of this study is to interrelate the crucial factors urban heat, vegetation cover, and socio-demographic/economic indicators by examining and analyzing geographical disparities and co-occurrences to inform spatial and urban planning for resilient and just cities.

## 2 The relationship between urban heat, urban green, and social status

The fact that cities feature higher temperatures than the surrounding countryside is presumably known since the first half of the 19th century [16]. According to Oke, the UHI is a thermal anomaly with vertical and horizontal dimensions, which's characteristics are found both in the intrinsic nature of the city (e.g., size/population, building density, land-use distribution) and external influences (e.g., climate, weather, seasons) [16]. The intensity of an UHI (UHII) is defined as the difference between rural and urban temperatures [8]. The (geographic) location, microclimatic influences, as well as background climate play an important role for the pronunciation of an UHI [e.g., 17,18]. Exemplary individual factors that cause and fuel UHIs are urban canyon geometry, air pollution, heat emission from buildings, traffic and living organism metabolism, as well as building materials [17]. The comprehensive set of factors that are of importance and that are researched intensively can be divided into two main groups: physical and social aspects of the urban composition or fabric. The former category tends to explain where and why UHI/heat hazard is most pronounced. The latter focusses on the exposure and vulnerability side, e.g., trying to find correlations between certain population groups and higher or lower exposure or vulnerability to the UHI (we follow the recent IPCC report for the definitions of, e.g., hazard, exposure, and vulnerability (with the sub-components sensitivity and adaptive capacity) in the risk framework [3]). A proxy often used to quantify UHIs, is the land surface temperature (LST), typically acquired airborne or with satellites [19,20]. One area of focus of this study are the spatial disparities of the vegetation provision and heat pronunciation (Chapter 2.1). Furthermore, our research contributes to two strands of urban environmental (in-)justice literature: analyzing the injustice regarding supply with urban green infrastructure (Chapter 2.2) and examining inequities in the thermal stress considering the socio-economic status of urbanites (Chapter 2.3).

### 2.1 Heat and green

The spatial distribution of UHI depends on morphological configuration, land use, land cover etc. While the entirety of land cover and land use is also intensely researched [21,22], the negative correlation between heat and vegetation is widely acknowledged and has been thoroughly described [[23], [24], [25]]. Here, the normalized difference vegetation index (NDVI) is often used as a proxy operationalizing vegetation cover and quality [e.g., 23,26].

### 2.2 Social factors and green supply

Especially in urban green infrastructure planning, we see a misbalance between social demand and social equity. US urbanized areas show less tree cover in low-income areas, which also tend to be hotter [27]. In Atlanta, African Americans have significantly poorer access to green spaces [28]. Various analyses have concluded that urban green is unevenly distributed in German cities, and both densely populated and socially disadvantaged districts are often inadequately supplied with urban green [29,30]. In addition, the studies show that socioeconomically well-off residents are predominantly found in areas with lower environmental stresses, while less privileged people are exposed to higher environmental stresses in their place of residence featuring higher health vulnerabilities at the same time [31]. In terms of policy action, the provision of green space in socially disadvantaged neighborhoods is particularly important. In such areas, the need for public green space tends to be higher due to the generally lower provision of private green spaces, which is further exacerbated by increased multiple pressures [[32], [33], [34], [35]].

### 2.3 Social factors and heat

Besides physical factors, a variety of socioeconomic and sociodemographic indicators are put in relation to heat. These are for example age, income, or race. Clear correlations between weaker societal classes and heat exposure are suggested by a large body of literature especially, but not exclusively, on US cities [5,8,23,[36], [37], [38]]. For Phoenix, Arizona, Buyantuyev and Wu [23] discover a weak but significant (p < 0.001) negative correlation (0.13–0.25) between income and UHI. Analyzing 20 Southwestern US metro areas, another study finds that, on average, the 10% poorest neighborhoods are 2.2 °C warmer than the most affluent 10%, representing an unequal exposure to heat [36]. Historic housing policies (redlining) persist in shaping inequalities also in climatic terms. Areas formerly impacted by redlining are found generally warmer than those not subjected to redlining [39,40]. People of color are also often located in areas with higher UHIs as proven by a study examining the 175 largest US urbanized areas [8]. Mitchell and Chakraborty researched the three largest US cities (New York City, Los Angeles, and Chicago) and detect lower economic status groups to be at higher heat risk [41]. In Philadelphia, however, Li does not find significant disparities in terms of race/ethnic groups, but elderly are found to live in cooler areas as well as high-income people [42]. The strong inequality effects found in research focusing on US study areas, according to Mitchell and Chakraborty, roots in the still present segregation. Accordingly, marginalized groups live in less desirable areas [6].

For other world regions, including Europe, there is not as much research to be found to date [Delhi, India: [37], Antwerp, Belgium: [43], Manchester, UK: [44]]. Burbidge et al. connect socio-economically marginalized communities, urban heat, and green space distribution in Antwerp, Belgium, and find heat injustice in so far that weaker social groups tend to live in areas that are less green and thus hotter [43]. In Manchester, UK, climate injustice could be determined as more diverse communities, people living in rent, and poor quality housing make up for a greater heat risk, while for elderly and children only a slight trend is found [44]. Another study compares the relationship between income and heat for 25 cities around the world. Here, 72% of poorer neighborhoods feature an elevated exposure to heat. Amongst other cities, the data for Berlin suggests that poorer households suffer from higher UHIIs [45]. Via a survey on German households, Osberghaus and Abeling, however, do not find differences in heat hazard and exposure for more or less deprived households [5].

Based on the reviewed literature, generally, one can say that socioeconomically well-off residents are predominantly found in areas with lower environmental stress, while socioeconomically disadvantaged are exposed to higher environmental stresses in their place of residence, with higher health vulnerability at the same time. Therefore, these neighbourhoods in particular should have a higher proportion of urban green space to compensate for the prevailing pressures such as pronounced heat. However, it has to be kept in mind that not only residential areas but also other places that people frequent, like the workplaces, where they spend a considerable amount of time, must not be excluded from a comprehensive vulnerability and exposure assessment.

### 2.4 Goals and RQs

The relationships outlined above are often regarded separately leading to the derivation of recommended actions based on the respective results. In the past, climate adaptation measures have also unintendedly led to an increase in climate injustice [3]. In order to avoid that, we follow a stringent integrated approach by regarding all the relationships between urban heat, vegetation, and social status, before combining the three factors in a cluster analysis. Such an approach is purposeful as, for example, the reduction of climate injustice and associated health issues are urgent tasks, for which not only the UHI distribution must be regarded but also the vegetation, especially in form of accessible and highly functional green areas. Thus, we examine the mentioned interplay in a post-industrial, segregated region subject to profound structural changes now and in the future. Our epistemic interest leads to the following research questions.

RQ 1 What does the relationship between heat and green provision look like?

RQ 2 What does the relationship between green provision and social status look like?

RQ 3 What does the relationship between heat and social status look like?

RQ 4 To what extent are spatial clusters disclosing and depicting similar heat, green supply, and social status conditions in the study area?

**Fig. 1:**
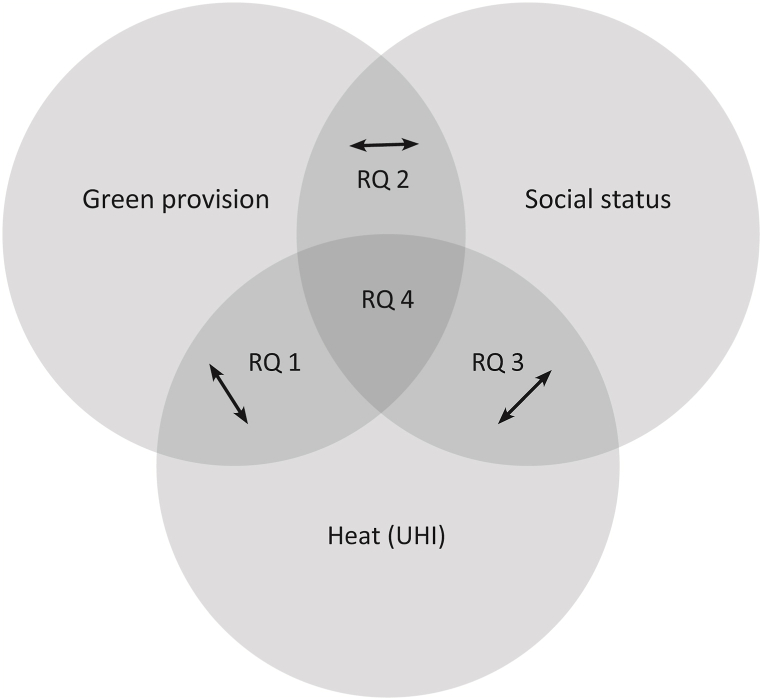
Graphical representation of the research questions.

While RQ 1, RQ 2, RQ 3 focus on the individual relationships between the factors regarded, RQ4 builds on these findings to combine the factors and to gain comprehensive insights on the interrelations and the spatial arrangement (see Fig. 1).

The remainder of the paper is structured as follows. The next section (Chapter 3) lists and explains the data and methodology applied in the course of this research. Then, Chapter 4 is dedicated to communicating and discussing the results obtained. Conclusions and an outlook complete this article in Chapter 5.

## 3 Material and methods

**Fig. 2:**
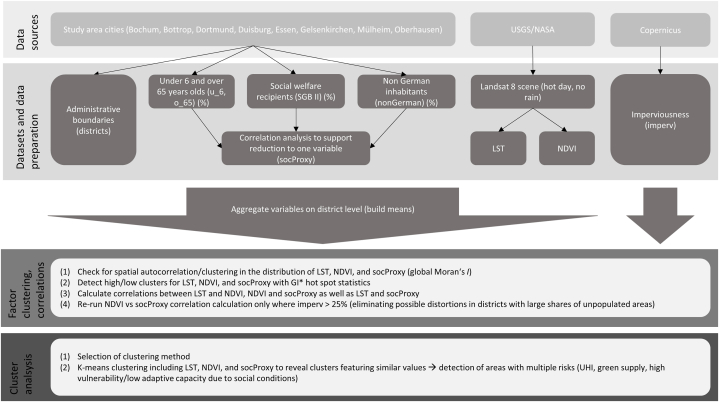
Methodological approach.

In order to answer the research questions, the following methodological approach, visualized in a research design (see Fig. 2), is applied. In a first step, the required data are procured and prepared accordingly. Subsequently, factors are correlated with each other. Finally, the factors are clustered to show underlying spatial structures of similarity and disparity. Preparing and analyzing the data is done with ArcGIS, GeoDa, and RStudio [[46], [47], [48]].

### 3.1 Study area

**Fig. 3:**
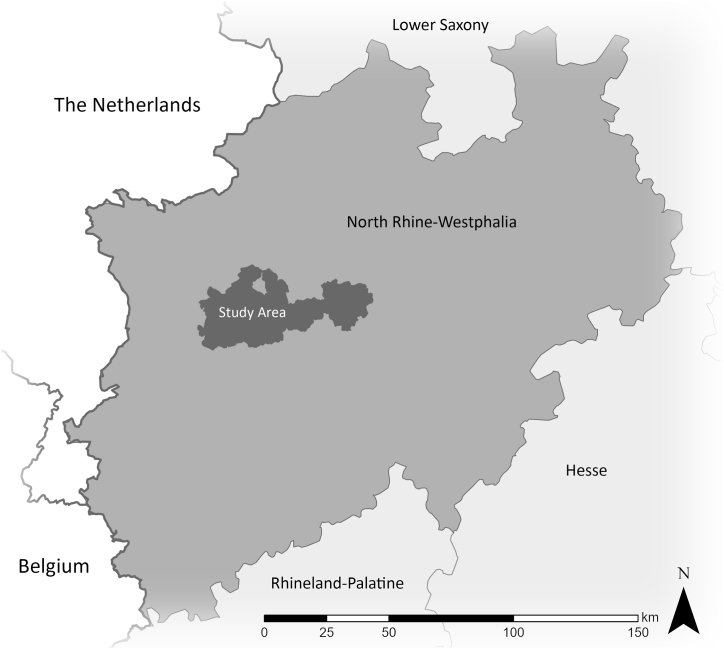
Location of the study area in North Rhine-Westphalia.

The research area for this study is the historically highly segregated Ruhr area in Western Germany (see Fig. 3), which is one of the largest metropolitan areas in Europe and densely populated. It is polycentric with a heterogeneous distribution of socio-spatial problems and economic potential. Therefore, it is most suitable for an evaluation of the relationships between heat, green provision, and social status factors. Inequalities in the Ruhr area arising from various historical development steps are particularly evident in a pronounced north-south divide along the federal highway A40 that runs through the whole region and is sometimes referred to as the social equator in both academia and the media [49,50]. It is crucial to note here that the A40 is not a cause but a symptom for the present segregation. The area north of the freeway, the so-called Emscher zone, was hit especially hard by the ongoing and intensifying structural changes as it was home to the majority of industrial workers [50]. The southern parts on the other hand, in the so-called Hellweg zone, where the industrialization took place earlier and that consists of existing older cities and settlement structures, had more time to restructure and adapt [51,52]. Describing this contrast, Wehling speaks of *organized complexity* in the Hellweg zone and of *disorganized complexity* in the Emscher zone [52]. Reflecting the south to north expansion of heavy industry in the Ruhr area, these structural heterogeneities are still perceptible [51]. Our study cities are Bochum, Bottrop, Dortmund, Duisburg, Essen, Gelsenkirchen, Mülheim, and Oberhausen as they all are situated along the mentioned A40. Some of these cities, like Dortmund and Essen, encompass districts in both zones featuring an internal north-south divide themselves while others, like Gelsenkirchen and Bottrop, are located completely in the northern Emscher zone displaying no such internal divide.

### 3.2 Datasets and data preparation

There are different approaches for capturing the spatial distribution of urban heat. UHIs and UHII are often operationalized by applying the LST as a proxy [20,22,37,53]. Today, Landsat 8 is adopted in various locations and with various temperature derivation methods [54]. To obtain LSTs representing the spatially differentiated heat hazard and thus also the exposure for people affected, we apply the algorithm presented by, amongst others, Avdan and Jovanovska [55] that is widely applied in the field [24,[56], [57], [58]]. For the aforementioned procedure Landsat 8 Bands 4, 5, and 10 are required. First, the thermal infrared Band 10 is used to derive the top of atmospheric (TOA) spectral radiance, which is then converted to the at-sensor brightness temperature (BT). Combining Bands 4 (red) and 5 (near-infrared), the NDVI is calculated [59], which serves as an input for the derivation of the proportion of vegetation. NDVI and proportion of vegetation are then used to determine the ground emissivity. Finally, the at-sensor temperature and the ground emissivity (as correction factor) are inputs for the final LST calculation. We choose a Landsat scene from a hot summer day in 2018 (maximum temperature above 30 °C [60]). As moisture plays an important role influencing the UHI [16], another inclusion criteria is *no precipitation for two days before the measurement*. Furthermore, we set the maximum cloud cover to 5%. Similar selection procedures are common in UHI research. Shandas et al., for example, chose data from days with maximum temperatures above the 90th percentile of historic averages [61]. Buyantuyev and Wu only included data from days prior to which there was no precipitation for four days and that were cloud-free [23]. The calculation of NDVI values, also part of the LST derivation above, as proxy for vegetation density/cover, and as such either mitigating or promoting heat exposure, is done using bands 4 and 5 of the said Landsat scene [59]. The Landsat 8 scene with its spatial resolution of 30 × 30 m for the LST and NDVI derivation comes from NASA's Earth Explorer platform [62].

There is wide range of socio-demographic and socio-economic factors that are applied describing vulnerability to heat of societal groups. One common variable is age. Here, very young and old people (often under 5/6 and over 65 years as a threshold) are considered more vulnerable to adverse heat effects [41,43,44,63]. In particular, the elderly group is suffering from the impacts of heat stress. Studies about previous heat waves have revealed that the morbidity and mortality rates of the elderly are increased during and post heat periods [64]. Thus, for this study we consider the share of the age groups under 6 (u_6) and above 65 (o_65) years as variables for age as vulnerability indicator.

Socio-economic status is operationalized with indicators like income [27,36,38,65], poverty [44], employment status [43], or social welfare reception [30,63]. Moreover, migration status [30,63], ethnicity/race [41,44,65], or minority membership [36,38] can be mentioned. Due to data availability and up-to-dateness, in our study, we use the social welfare reception (SGB II) and the nationality status (nonGerman) as indicators for the socio-economic status contributing to vulnerability. Unfortunately, there is no free and high resolved data on health status being a factor determining vulnerability. However, one motivation for our research are the potential effects on health that excess heat combined with a low green provision can have on vulnerable groups.

Administrative boundary data (statistical districts) as well as socio-economic and socio-demographic data on age groups, social welfare recipients (SGB II) and nationality status (nonGerman) is obtained from the cities regarded [[66], [67], [68], [69], [70], [71], [72], [73]]. For Dortmund, the reporting date is 12/31/2019, for all other cities it is 12/31/2021 for age data and 12/31/2020 for SGB II and nonGerman. In total, we analyze 275 districts in this research. Three districts in Duisburg could not be included due to insufficient data availability for the social indicators.

For the correlation analyses between heat, vegetation, and social factors, we fathom the possibility of combining or reducing the social factors without losing substantial informative value. To do so, we calculate correlations between the social factors mentioned above for the whole study area and aggregated to the cities within.

**Appendix Fig. 1:**
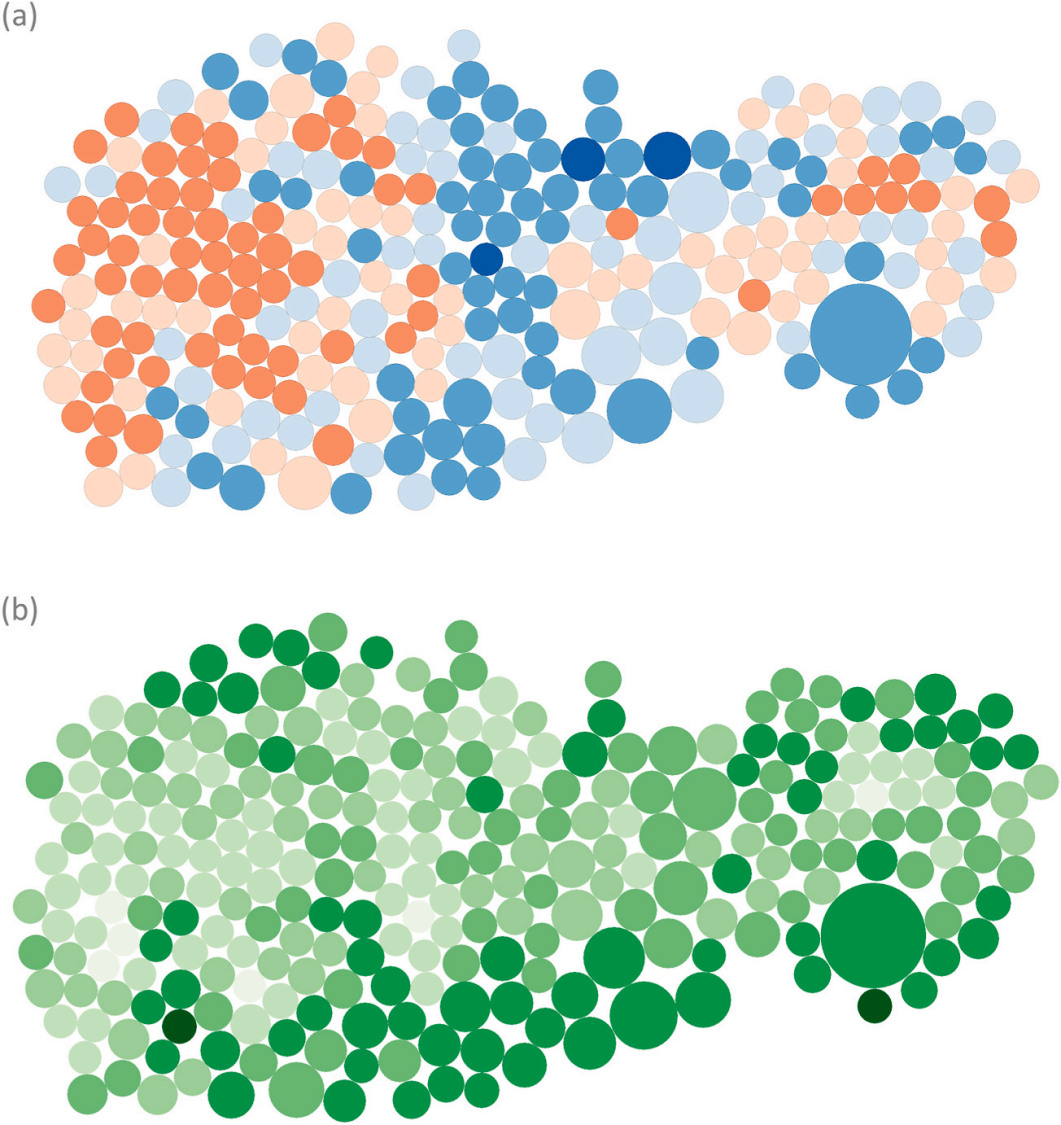
Cartograms depicting urban heat (LST) (a) and NDVI (b) respectively in combination with the population older than 65 years on a city district level. Circle size represents the share of people over 65 years. Red coloring describes higher LSTs, blue coloring lower LSTs (a). The intensity of the green coloring stands for a higher NDVI (b).

Table 1 shows that relating u_6 and SGB II with the indicator nonGerman features high positive correlation coefficients. Between o_65 and nonGerman, the correlation coefficient proves to be negative (nonGerman population does not coincide with high shares of elderly). Nevertheless, we decide for nonGerman as our single social status indicator/proxy. Our approach focusses rather on the relationships between socially deprived populations and LST as well as NDVI than on urbanites’ vulnerability in general. Regarding elderly persons (o_65) there is evidence that, while their propensity to be adversely affected regarding health issues is indisputable (see above), they are often not exposed to heat to a higher degree. For instance, in a study considering Philadelphia this was found by Li [42]. Data for our study area also supports these findings. The cartograms provided in Appendix Fig. 1 reveal, that elderly inhabitants predominantly live in cooler and greener regions of the study area. Thus, excluding the age indicators as standalone (elderly nonGerman people are still covered) variables is viable for our purposes. Especially its very strong correlation to the social welfare quota (SGB II) makes nonGerman a suitable indicator that, in addition to representing probable social weakness, also covers potential language barriers people might face. Thus, the non-German population can also be seen as more prone to the risk of heightened heat exposure and especially vulnerability regarding adverse health effects connected with urban heat. Although, in the recent past, substantial shares of the non-German population originate from countries with warmer climates potentially featuring both a lowered level of sensitivity and an increased knowledge regarding adaptation strategies, their often precarious economic situation (see correlation with SGB II quotas) prevents them from financially and factually being able to put in value these experiences (e.g., change the place of residence or making adjustments to their homes). While for our aggregation level (districts), nonGerman as a proxy works well for the reasons mentioned above, detailed analyses on a higher resolved spatial level might certainly require other additional factors.

**Table 1:** Correlation (r-values) between social factors in the whole study area and for Bochum (BO), Bottrop (BOT), Dortmund (DO), Duisburg (DU), Essen (E), Gelsenkirchen GE), Mülheim (MH), and Oberhausen (OB). *** significant at 0.001 level, ** significant at 0.01 level, * significant at 0.05 level.

Variables		1 u_6	2 o_65	3 SGB II	4 nonGerman
**1 u_6**	All cities	1			
	BO	1			
	BOT	1			
	DO	1			
	DU	1			
	E	1			
	GE	1			
	MH	1			
	OB	1			
**2 o_65**	All cities	−0.595***	1		
	BO	−0.436*	1		
	BOT	−0.738***	1		
	DO	−0.478***	1		
	DU	−0.758***	1		
	E	−0.684***	1		
	GE	−0.743***	1		
	MH	−0.779***	1		
	OB	−0.768***	1		
**3 SGB II**	All cities	0.373***	−0.554***	1	
	BO	0.712***	−0.692***	1	
	BOT	0.706**	−0.326	1	
	DO	0.794***	−0.588***	1	
	DU	0.781***	−0.854***	1	
	E	0.754***	−0.817***	1	
	GE	0.499*	−0.163	1	
	MH	0.703***	−0.853***	1	
	OB	0.873***	−0.788***	1	
**4 nonGerman**	All cities	0.704***	−0.681***	0.554***	1
	BO	0.538**	−0.887***	0.841***	1
	BOT	0.721**	−0.440	0.964***	1
	DO	0.684***	−0.644***	0.889***	1
	DU	0.810***	−0.903***	0.925***	1
	E	0.604***	−0.886***	0.848***	1
	GE	0.656**	−0.306	0.918***	1
	MH	0.708***	−0.811***	0.916***	1
	OB	0.999***	−0.732***	0.939***	1

As some, especially peripheral, districts feature only small built-up, developed areas and are otherwise dominated by agricultural land or forests, we repeat the NDVI vs nonGerman correlation calculation (described in Chapter 3.4) with a modified setup including impervious surface data (imperv). The imperv data used is in a raster resolution of 10 × 10 m and stems from the Copernicus database (reporting date 2018) [74].

### 3.3 Descriptive stats and factor distribution in the area

For all variables applied (LST, NDVI, nonGerman) we determine the mean for each district (aggregation). As each aggregation procedure comes with a certain bias, we furthermore calculate the coefficients of variation in the distinct districts to better embrace the situation within the neighborhoods. This also helps interpreting and describing the results from the following cluster analyses. The next step is the calculation of basic stats (minimum – min, maximum – max, mean, median, standard deviation – sd) for each district for LST, NDVI, and the social indicator nonGerman.

We conduct a global Moran's *I* analysis (clustered vs random distribution) and Gi* calculations (reveal locations of high/low value clusters) to examine whether the indicators regarded are clustered and not randomly distributed in the study area [75]. For both processes we apply a queen contiguity (all neighbors sharing a border with the unit regarded are part of the neighborhood) based approach to model the neighborhood and to get a spatial weights matrix ($W=\left(w_{ij}\right)$) for the pairwise comparison of spatial units. The *I* values are to be interpreted including the respective p-values and generally reach from −1 (negative autocorrelation) to 1 (positive autocorrelation), with values close to 0 meaning no autocorrelation. The formulas for the Gi* hot spot statistics as well as the global Moran's I are given below:

(1) $$\begin{array}{c} G_{i}^{*}=\frac{\sum_{j}^{n}w_{ij}∙x_{j}}{\sum_{j}^{n}x_{j}}, \end{array}$$

*with* n *being the number of spatial units (districts), and* $x_{i}$*,* $x_{j}$ *the attribute values at locations* i *and* j*.*

(2) $$\begin{array}{c} I=\frac{n}{\sum_{i,j}^{n}w_{ij}}∙\frac{\sum_{i,j}^{n}w_{ij}\left(x_{i}-\bar{x}\right)\left(x_{j}-\bar{x}\right)}{\sum_{i}^{n}{\left(x_{i}-\bar{x}\right)}^{2}}, \end{array}$$

where n is the number of spatial units (districts), $\bar{x}$ denotes the average of the observed attribute values, and $x_{i}$, $x_{j}$ are the values at locations i and j.

### 3.4 Correlation analysis

The answers to RQ 1, RQ 2, RQ 3 are generated with correlation analyses for the whole study area as well as for the distinct cities therein. For RQ 1 we calculate the correlations (Pearson) between LST and NDVI, for RQ 2 the same is done for the relationship between NDVI and nonGerman, and for RQ 3 finally, we look at LST and nonGerman. In order to eliminate possible distortions in districts with large shares of unpopulated areas, we re-run the analysis with NDVI and nonGerman only where the imperviousness (imperv) is over 25% suggesting an urban structure [76]. Data on population densities as a measure for the presence of people is unfortunately not available in spatial resolutions sufficient for our purposes.

### 3.5 Cluster analysis

Building on the previous findings and based on RQ 1, RQ 2, RQ 3, our final research question (RQ 4) is dedicated to the detection of areas (district clusters) that feature similar indicator values and can thus describe multiple issues: UHI and overheating through high LSTs, issues with green supply (NDVI), and heightened vulnerability or low adaptive capacity due to social conditions (nonGerman). In a cluster analysis, the allocation algorithms serve the aim of minimizing the variability of the spatial units within a cluster and at the same time maximizing the variability between the clusters. Only by this, generalizable statements about spatially differentiated strategies are possible. In our case, cluster formation is based on the characteristics of the three factors UHI, NDVI and nonGerman. With such a large number of cases (n = 275), a suitable number of clusters is usually first searched for, and, in a further step, the cases are (re-)assigned to the clusters – the procedure therefore consists of two steps.

1. Hierarchical cluster analysis (Ward algorithm; optimization of squared Euclidean distances) with the previously determined factor values [77]. The aim is to determine the optimal number of clusters and the cluster centers (average values of the factor values in the districts belonging to the cluster).
2. Cluster center analysis (k-means) with the factor values [78]. The aim is to optimize the cluster affiliation of the statistical districts based on their distance from the cluster center.

By means of hierarchical (agglomerative) cluster analysis, the districts with the smallest Euclidean distance (determined based on the factor values for LST, NDVI, and nonGerman) are grouped together. Ward's method is utilized for the clustering. This method is based on the distance between the respective value and a central point in each cluster, which tends to result in nicely balanced clusters.

Thereafter, the cluster centers are determined for the respective clusters provided in step one. In addition to the number of clusters, these are required to enable the best possible allocation of the statistical districts to clusters based on the cluster center analysis. The cluster center represents the combination of the mean values of the characteristics of the three factor values. In practice, an ideal hypothetical district is formed, representing the center of a cluster.

The k-means algorithm is based on the squared Euclidean distance as the measure of dissimilarity. The districts with corresponding factor values are assigned to the cluster centroid to which they are closest, using a Euclidean (squared difference) dissimilarity criterion. The k-means method uses an iterative relocation heuristic as the optimization strategy. This means that after an initial solution is established, subsequent moves (i.e., allocating observations to clusters) are made to improve the objective function. At each step, the total of the within-cluster sums of squared errors (from the respective cluster means) across all clusters is lowered.

## 4 Results and discussion

### 4.1 Descriptive stats, global autocorrelation, and factor clustering

Table 2 depicts the basic descriptive statistics for LST, NDVI, and nonGerman for the total study area as well as for the specific cities therein. No peculiarities in the data can be seen here. Cities with more districts (higher n) feature greater differences between min and max than cities with lower n. However, the mean and median values are always close together signalizing the lack of outliers. The same is true for sd values that are all in the same range for the cities and indicators regarded.

**Table 2:** Basic stats regarding LST, NDVI, and nonGerman for every study city and all cities together.

Variables		LST [°C]						NDVI			nonGerman [%]				
Spatial unit	Min	Max	Mean	Median	sd	Min	Max	Mean	Median	sd	Min	Max	Mean	Median	sd
**All cities (n=75)**	23.60	32.90	28.98	29.17	1.75	0.08	0.43	0.25	0.25	0.05	2.10	60.15	17.10	14.08	11.55
**BO (n=30)**	23.60	30.34	28.12	28.52	1.60	0.13	0.34	0.26	0.26	0.04	3.13	33.21	14.67	11.78	7.85
**BOT (n=17)**	25.92	31.29	29.22	29.14	1.56	0.11	0.40	0.27	0.27	0.05	2.80	27.4	11.09	10.80	7.47
**DO (n=62)**	24.92	31.38	28.97	29.14	1.26	0.11	0.40	0.27	0.27	0.05	3.65	59.59	15.73	12.30	13.75
**DU (n=43)**	26.32	32.10	30.21	30.29	1.27	0.08	0.31	0.21	0.23	0.05	4.49	60.15	22.10	18.75	13.75
**E (n=50)**	22.48	31.68	28.12	28.36	1.69	0.10	0.35	0.26	0.26	0.05	2.20	50.60	16.53	14.40	11.23
**GE (n=18)**	24.85	29.01	26.85	26.93	1.29	0.17	0.33	0.25	0.26	0.04	7.23	43.65	24.15	23.55	11.60
**MH (n=28)**	24.79	32.90	29.62	29.63	1.46	0.10	0.43	0.25	0.26	0.07	2.10	46.60	15.75	13.40	11.64
**OB (n=27)**	26.63	32.33	30.19	30.42	1.60	0.13	0.35	0.23	0.22	0.05	2.70	37.30	16.49	15.50	8.58

**Appendix Fig. 2:**
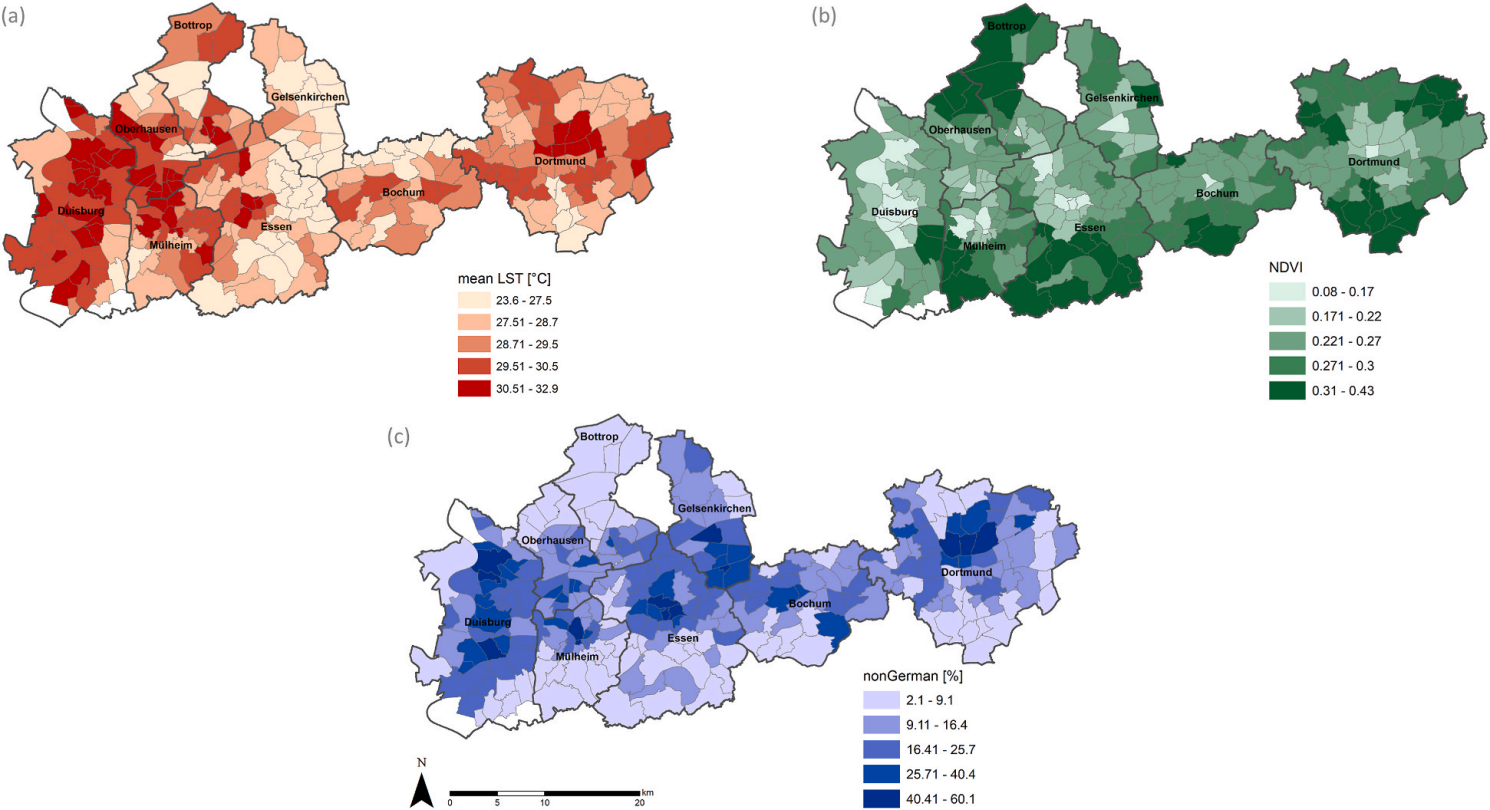
City district means for LST (a), NDVI (b), and nonGerman (c).

**Appendix Fig. 3:**
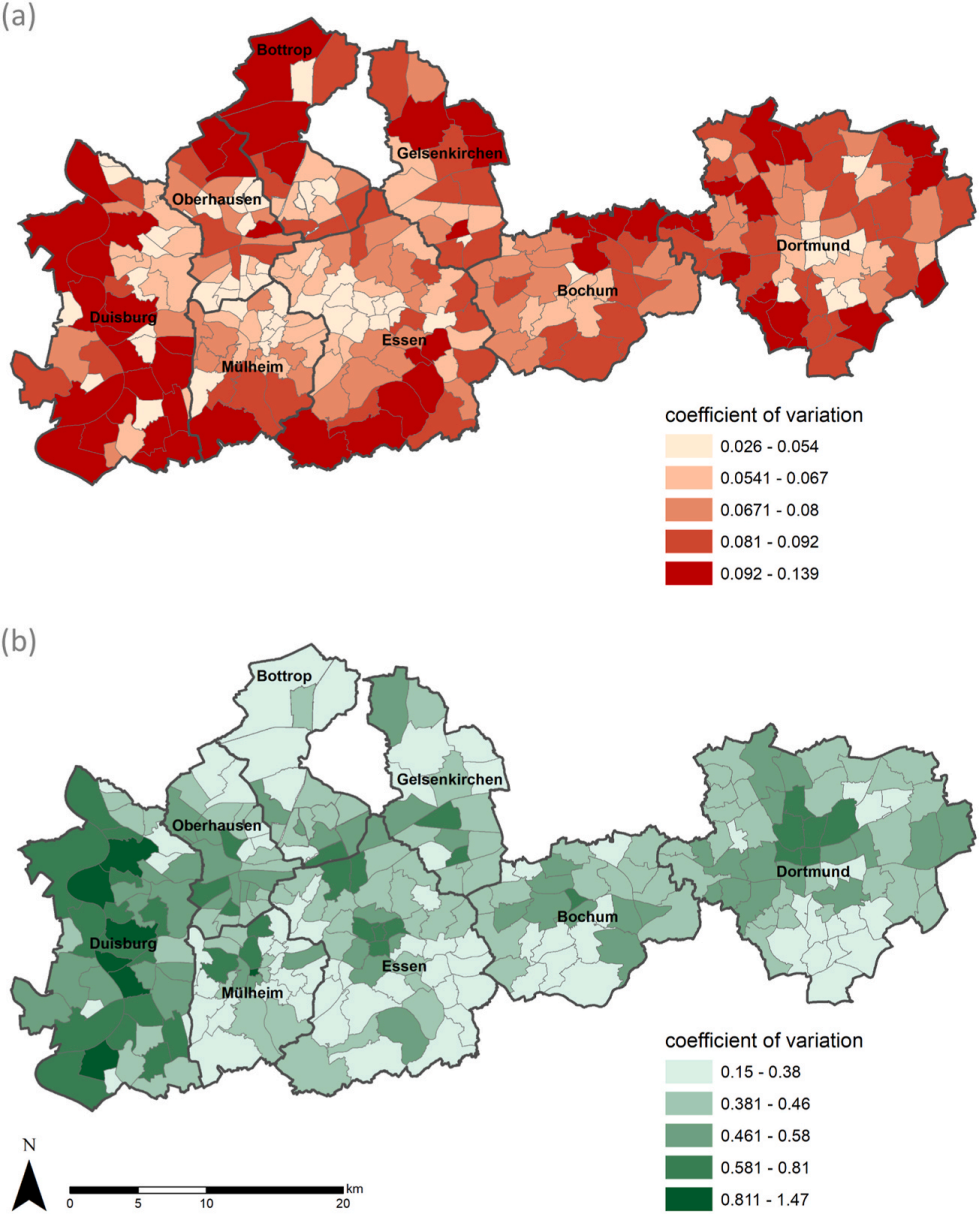
Coefficients of variation for LST (a) and NDVI (b) on the city district level.

As supporting material (e.g., for the intepretation of clusters later on), we provide maps depicting the result of the mean calculation for each district and each of the three parameters applied in Appendix Fig. 2. Furthermore, Appendix Fig. 3 contains two maps showing the coefficients of variation for LST and NDVI on the district level. As nonGerman data was obtained on the (politically relevant) district level without any information on the variation below this spatial level, no coefficient of variation calculation could be conducted.

**Fig. 4:**
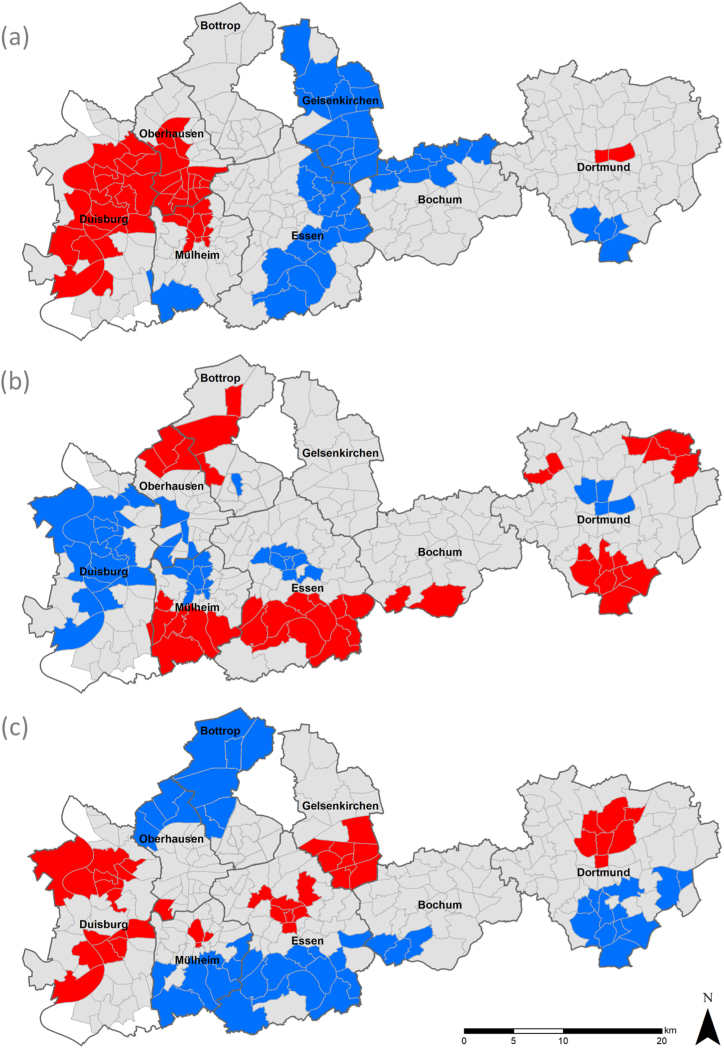
Cluster analysis (high-low clusters) with Gi* statistics on the distribution of LST (a), NDVI (b), and the share of non-German inhabitants (c) in the districts of the study area. Red stands for high value clusters, blue for low value clusters.

Moran's *I* for LST lies at 0.6, for NDVI it is 0.5, and for the share of non-German inhabitants *I* is 0.47. All three values suggest spatial autocorrelation and thus clustering of similar values in the same region. This assumption is further confirmed by the results of the Gi* cluster analyses. Fig. 4 shows high-low clusters (Gi*) on the district level for LST (a), NVDI (b), and nonGerman (c). Clusters depicted are at least significant on the 95% confidence level and are the result of 999 permutations. On the level of the whole study area, high temperature clusters are found in the densely built and populated northern inner city districts of Dortmund and large parts of Duisburg and Oberhausen in the west. Cooler districts are found in the rural south of Dortmund and Mülheim as well as in the north of Bochum and almost all districts of Gelsenkirchen and the eastern districts of Essen. High NDVI values and thus a higher vegetation cover cluster in many of the norther- and southermost parts of the Ruhr area where more districts with rural spatial structures dominate. Least green areas on the other hand are found in inner city districts that are often characterized by a high level of impervious surfaces and a lack of green/blue infrastructure. The inner/outer city contrast is even more pronounced when the share of nonGerman is regarded. High clusters are found in central city parts, whereas the most distant, rural districts feature the lowest shares and form low clusters.

### 4.2 Correlation analysis

**Fig. 5:**
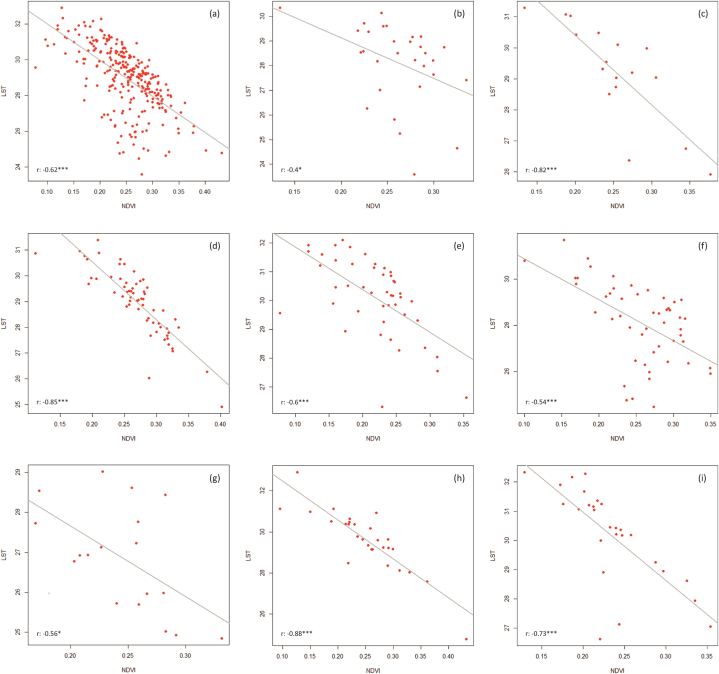
Scatter plots (LST vs NDVI) with linear regression lines for the whole study area (a) and the cities of Bottrop, Bochum, Dortmund, Duisburg, Essen, Gelsenkirchen, Mülheim, and Oberhausen (b–i).

**Fig. 6:**
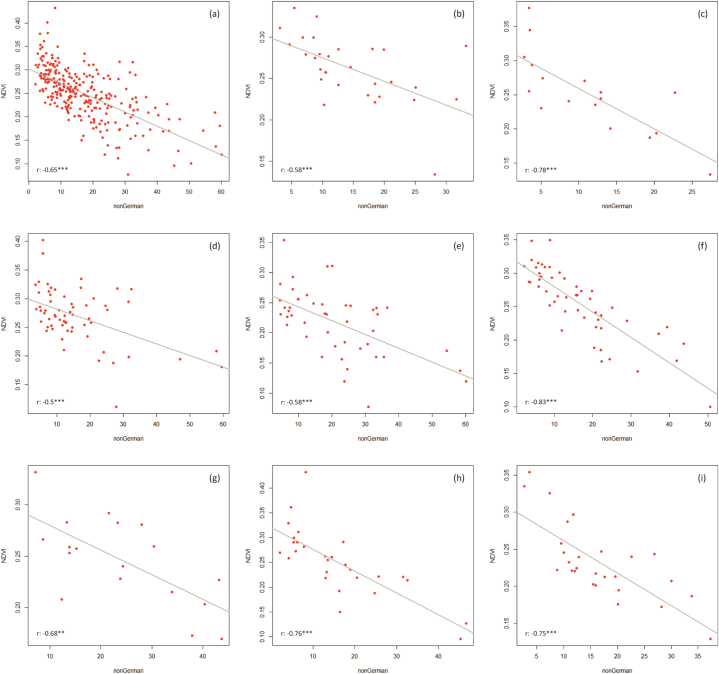
Scatter plots (NDVI vs nonGerman) with linear regression lines for the whole study area (a) and the cities of Bottrop, Bochum, Dortmund, Duisburg, Essen, Gelsenkirchen, Mülheim, and Oberhausen (b–i).

**Fig. 7:**
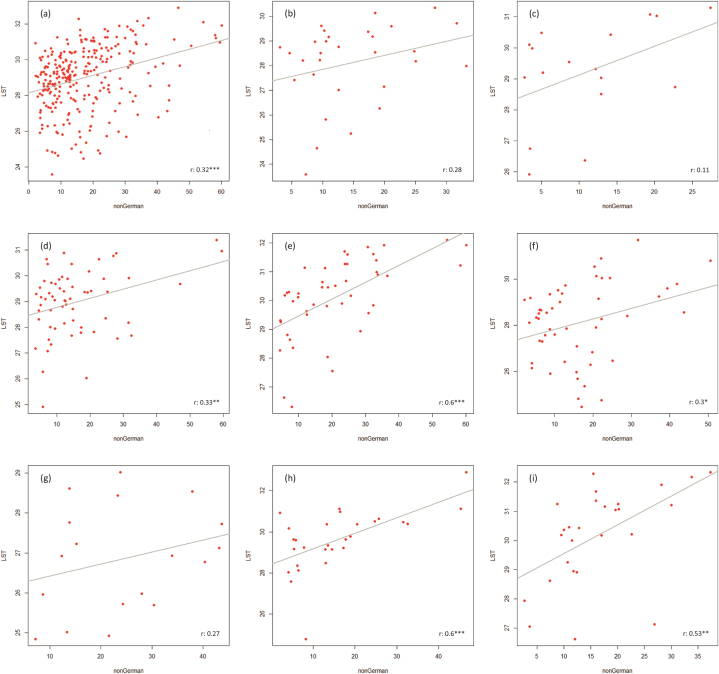
Scatter plots (LST vs nonGerman) with linear regression lines for the whole study area (a) and the cities of Bottrop, Bochum, Dortmund, Duisburg, Essen, Gelsenkirchen, Mülheim, and Oberhausen (b–i).

Fig. 5, Fig. 6, Fig. 7 show the correlation results for the combinations LST vs NDVI (Fig. 5), NDVI vs nonGerman (Fig. 6), and LST vs nonGerman (Fig. 7). The LST vs NDVI case (RQ 1) shows correlation coefficients reaching from −0.4 (Bochum) to −0.88 (Mülheim). All correlation results are significant on the 0.001 level except for Bochum and Gelsenkirchen (0.05). The relationship is negative for all cities. This is also what was expected from previous research (see Chapter 2.1).

The NDVI vs nonGerman (RQ 2) shows negative correlation coefficients from −0.5 (Dortmund) to −0.83 (Essen). Here, the assumed relationship between weaker societal status and lower vegetation cover is confirmed (see Chapter 2.2). Except for Gelsenkirchen (0.01) all correlations are significant on the 0.001 level. The differences between cities, however, are quite large. Dortmund obviously features green spaces also in areas inhabited by less Germans. Whereas the opposite is true for Essen, where less nonGermans live in green areas. The re-run of the NDVI-nonGerman correlation analysis features very similar results compared to the original calculation without the imperviousness restriction. This, at first sight somewhat surprising outcome, can be comprehended by looking at the land use/land cover structure of the districts. Low built-up shares mainly occur in rather peripheral districts where the building density and population density generally is lower than in central parts. Thus, restricting the NDVI vs nonGerman analysis to only these zones does not change the previously perceived relationship. Furthermore, the social proxy (nonGerman) regularly features higher values in more central parts of urban areas (due to assumed employment opportunities, clustering of functions, and higher availability of living space, e.g.).

When it comes to the LST and nonGerman relationship (RQ 3), the correlation analysis shows an ambiguous picture. Bottrop features a very low correlation coefficient of 0.11, while the highest one is found in Duisburg and Mülheim (0.6). For Bochum, Bottrop, and Gelsenkirchen, the correlation results are not significant, for Essen it is significant on the 0.05 level, for Dortmund and Oberhausen on the 0.01 level, and for Duisburg and Mülheim on the 0.001 level. Non-German citizens are thus heterogeneously impacted by higher temperatures in our study area. These diffuse outcomes match previous studies’ findings applying similar indicators, according to which for some cities strong correlation and injustices were found [36,43] and for others this was not the case [5,42]. This reinforces the need for detailed analyses. We can conclude that the relationship between our social proxy (nonGerman) and heat is not area-wide significant and strong, but it is for some cities and possibly also their respective neighborhoods. Our results can serve as first indication for the need of future investigation of certain areas.

### 4.3 Cluster analysis

**Fig. 8:**
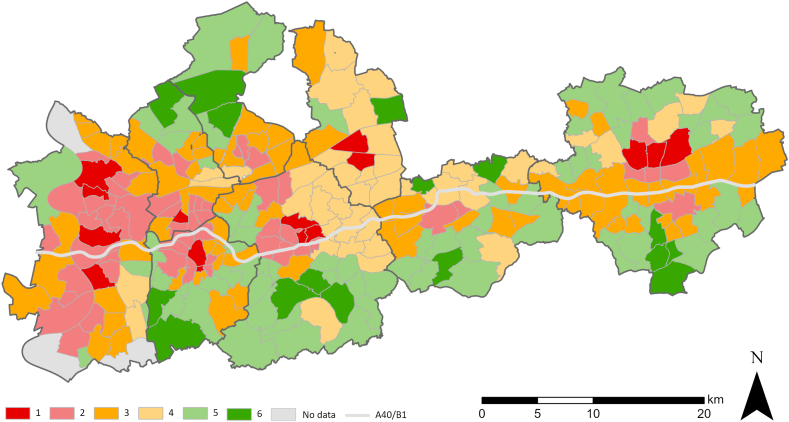
Cluster analysis results.

Building on the previous correlation analyses and in order to gain more profound insights on the interplay of all three factors we conduct a cluster analysis (RQ 4). This helps visualizing the distribution of UHI taking into account the social structure and green space provision to indicate the need for action. The set number of clusters determined by the hierarchical cluster analysis is six. Fig. 8 shows the spatial distribution of the six clusters. Underlying factors like the historical development, spatial structure or building density of the districts can serve as potential explanations for the resulting clustering. The following six types of clusters can be differentiated:

Cluster 1: districts with high temperatures and high proportions of socially deprived groups, with very low green provision → highly concentrated city center locations, often in the Emscher zone.

Cluster 2: districts with high temperatures and low green provision, but less socially deprived groups → densely built-up and sealed inner city locations.

Cluster 3: districts with relatively high temperatures and low green provision, but significantly less socially deprived groups → peripheral city areas.

Cluster 4: districts with significantly lower temperatures and higher green provision, but still high proportion of socially deprived groups → peripheral areas, mostly in between cities.

Cluster 5: districts with low temperatures and a high green provision, as well as low proportion of socially deprived groups → peripheral areas with a rural spatial structure.

Cluster 6: districts with the lowest temperatures and highest green provision as well as lowest proportion of socially deprived groups → peripheral areas with rural spatial structures.

In addition to the mean values of the three factors (LST, NDVI, nonGerman) within the clusters, Table 3 shows the number of districts in the clusters – in each case as a result of the hierarchical cluster analysis (step 1) and after re-sorting as part of the cluster center analysis (k-means). Changes occur in all those districts which's distance to another cluster center is less than the original ‘own’ center in step 1. Comparing the mean values with each other, one can see the differences of the clusters. There are two clusters (cluster 1 with 30.57 °C and cluster 2 with 30.8 °C) with very high values for the factor LST, but both clusters differ significantly in the case of the social factor nonGerman (cluster 2 with 25,66% and cluster 1 with 47.7%).

**Table 3:** Mean factor values within the clusters.

Nr.	LST [°C]	NDVI	nonGerman [%]	Number of districts (step 1)	Number of districts (step 2)
1	30.57	0.15	47.70	30	15
2	30.81	0.19	25.66	81	54
3	29.68	0.24	13.11	41	78
4	26.61	0.26	21.20	60	43
5	28.56	0.29	8.33	47	69
6	26.03	0.35	5.75	16	16
Total				275	275

**Fig. 9:**
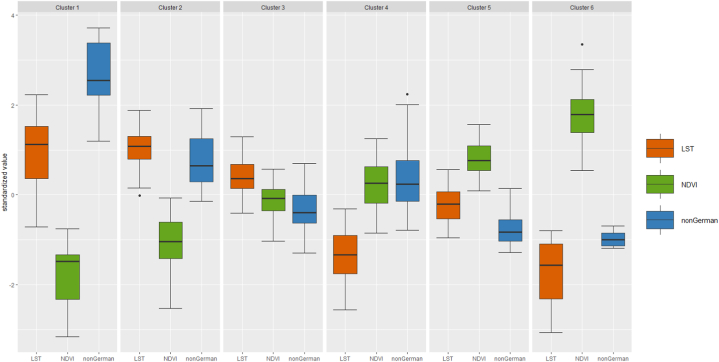
Boxplots for the individual clusters.

Table 4 shows the sd and variance with respect to the distances to the cluster centers within each cluster. In addition to the box plot graphs (Fig. 9), these statistical indicators provide information on how homogeneous a cluster is. While cluster analyses try to minimize the differences within the clusters, there are always outliers, which are not very similar to any cluster center. The final cluster assignment shows that there are more homogeneously occupied clusters with a small dispersion within the cluster and more heterogeneously occupied clusters with a larger dispersion (Table 4).

**Table 4:** Distances to the cluster centers: standard deviation (sd) and variance.

Nr.	Number of districts	sd LST [°C]	Variance LST [°C]	sd NDVI	Variance NDVI	sd nonGerman [%]	Variance nonGerman [%]
1	15	1.54	2.36	0.040	0.0016	8.98	80.64
2	54	0.74	0.54	0.032	0.0010	6.31	39.83
3	78	0.69	0.47	0.018	0.00003	5.15	26.53
4	43	1.08	1.17	0.029	0.0009	8.28	68.52
5	69	0.65	0.42	0.019	0.0004	4.14	17.17
6	16	1.17	1.37	0.036	0.0013	2.00	4.00
Total	275						

Through the box plots (Fig. 9), it becomes apparent that cluster 1 is very heterogeneous. The statistical findings in Table 4 underline that: with a standard deviation of 8.98% (nonGerman), 1.54 °C (LST), and 0.04 (NDVI), cluster 1 is by far the most heterogeneous, possibly despite or precisely because of the small number of districts (15) in this cluster. Cluster 3 and 5 are remarkably homogeneous – again despite or precisely because of the large number of districts (78 and 69). The same applies for cluster 4, where there are far fewer districts (43). With standard deviations of 2.00% (nonGerman), 1.17 °C (LST) and 0.0013 (NDVI), the dispersion is low and well below the average at least for the values nonGerman and NDVI (standard deviation of 2.61% and 0.009). For in-depth analyses and interpretation, the maps depicting the coefficients of variation for LST and NDVI on the district level (Appendix Fig. 3) provide valuable additional information. Regarding heat (LST), the variation is generally rather low, reaching a maximum value of about 0.14. Especially warmer, central districts seem to be rather homogeneous regarding LST values, as they feature lower coefficients of variation compared to cooler, more peripheral areas. NDVI coefficients of variation on the other hand are generally much higher, reaching a maximum of 1.47. Roughly speaking, the occurrence of high/low coefficients is inverted compared to the LSTs. Highest NDVI variations are found in the rather central, warmer areas, lower values are often in cooler, peripheral neighborhoods. While the warmest areas seem to feature rather homogeneous temperature regimes due to, e.g., a high general level of imperviousness and building density, the immediate proximity between sealed and unsealed surfaces (parks, gardens etc.) leads to high variations regarding the local NDVI. Cooler, peripheral areas with more mixed and balanced land uses feature more distinguished LST regimes, which results in higher coefficients of variation. NDVI variation is lower especially in districts that are predominantly green with only scattered settlement structures.

The cluster analysis on the aggregation level of statistical districts (Fig. 8) shows that the districts, in which a particularly large number of non-Germans live are mostly concentrated north of the A 40 in the Emscher zone, which is persistent compared to studies using older data [[50], [51], [52]]. The disparities on the green provision also mirror the (rough) bipartite division of the study area. The inner city-districts, with high densities, and the former old industrial areas with corresponding former old worker's housing estates north of the A40 contain less green spaces. These districts also show higher temperatures. Urban green as a factor in climate adaptation and mitigation against heat stress is not distributed according to the population's needs. Green spaces tend to be least available in districts with a higher share of socially deprived groups. These districts often have an increased need for attention, due to the high density of settlements and their social structure. A closer look at the population distribution in these districts shows that 27.01% (744,659 people) of the population (Table 5) in the case study cities of the Ruhr area live in districts (cluster 1 and 2) that are characterized by high temperatures, low green provision, and a high proportion of socially deprived groups. Only 24.2% (665,397 people) (Table 5) live in districts (cluster 5 and 6) with low temperatures and a high vegetation coverage.

**Table 5:** Population distribution of the clusters.

Nr.	Number of districts	Proportion of residents	Number of residents
1	15	6.12%	170,580
2	54	20.89%	574,079
3	78	29.39%	807,721
4	43	19.31%	530,756
5	69	20.73%	569,889
6	16	3.47%	95,508
Total	275	100%	2,748,533

The cluster assignment across the analyzed cities also shows a heterogeneous distribution of the clusters within the cities (s. Fig. 8). Dortmund and Bochum have a more circular historical spatial structure, with, on the one hand densely built-up inner-city districts with prevailing pressures (cluster 1 and 2) and, on the other hand more peripheral districts with rural spatial structures and less heat stress (cluster 5 and 6). Duisburg and Essen as well as Gelsenkirchen have a linear structure, where peripheral districts with rural structures characterized by lower building density and a higher vegetation coverage are located south or north of the city center. Overall, in all cities, the inner-city districts can be seen as hot spots. A closer look at cluster 4 discloses some of the limitations of the aggregation and clustering at district level. Due to the underlying variation of the variables for heat and vegetation coverage, cluster 4 becomes harder to interpret. This cluster consists of districts with significantly lower temperatures and higher green provision, but still high proportion of socially deprived groups in relation to the entire study area. For example to explain the appearance of a “cold belt” in the Emscher zone that encompasses basically the whole city of Gelsenkirchen, more information is needed. The districts of Gelsenkirchen often feature both dense building structures and green areas. On the district aggregation level, these two variables can balance each other suggesting generally less pronounced urban heat effects and thus less issues due to heat stress in these districts. Appendix Fig. 3 shows rather high LST coefficients of variation for Gelsenkirchen suggesting the presence of heterogeneous heat burdens. On a more detailed level potential local hot spots have to be detected in order to inform and guide tailored adaptation measures. Another aspect to be considered is the character of areas that are for example very hot. In the north of Essen there are cluster 2 areas that consist mainly of industrial land uses and not mixed/residential structures as in other cluster 2 regions. While it is important to know that for the selection and prioritization of adaptation action, a focus only on residential areas in order to counter adverse heat effects, such as health impacts, is too narrow, as depending on daytime and phase of life, whereabouts of people are very diverse.

Nevertheless, the analysis shows that, on a level relevant for urban planning, there are spatial clusters depicting similar UHI, green provision, and social status in the study area. Our results represent an addition to the well-described three dimensions of segregation, namely social, demographic, and ethnic, present in the Ruhr area, which are the result of the economic history and thus also land use changes [[50], [51], [52]], by further considering disparities and co-occurrences regarding urban heat and urban green provision. It becomes apparent that there are districts with an urgent need for action regarding the three factors considered. However, there are also districts with less heat stress due to spatial structures and less socially deprived groups. A closer look at the differentiation of the districts shows that each cluster of districts has its own interplay of UHI, NDVI, and nonGerman. Visualizing and analyzing these differences allows specific measures for adaptation and mitigation of heat stress as well as addressing climate injustice in the cities of the Ruhr area.

### 4.4 Limitations

The data used and the chosen methodological approach exhibit certain limitations. In the German context, data availability, especially on the social status and socio-demographic factors, is unfortunately insufficient in parts, in particular when it comes to high resolution data, therefore not all relevant aspects can be covered with suiting data (e.g., income or health data). Furthermore, more fine-grained data on all ends (LST, NDVI/vegetation coverage, social indicators) would allow for more detailed aggregation levels than districts, potentially exposing different impact and distribution patterns. Due to, in parts strongly, varying district sizes and internal structures, the aggregation via means might bias results. We counter that by additionally calculating the coefficient of variation on the district level allowing inferences on the extent of variability in relation to the mean of the respective variable values. However, more sophisticated normalization procedures might further enhance the transparency and comprehensibility of results. Moreover, temperature and green provision information can probably be made more robust by combining multiple scenes for an average see, e.g., [79]. Finally, combining data stemming from different sources or featuring various spatial resolutions is challenging and a potential source for uncertainties due to the aforementioned necessity of aggregation and the need to compromise regarding the temporal and spatial accuracy of fit.

When it comes to methods, the correlations (especially when n is small) are quite sensitive to outliers leading to misinterpretations. The task for planners and administration is thus to check distributions in detail. In order to determine the influences of certain factors on heat, regression analyses are a future step. Longitudinal approaches, e.g., comparing the last ten years might reveal trends and give hints for future developments, too. Results might also look different when not only social vulnerability is included but health vulnerability in particular or vulnerability as a whole.

The different methods of clustering usually yield very different results. This occurs because of the different criteria for merging clusters (including cases). K-means has trouble clustering data tending to from clusters of varying sizes and densities. Centroids can be dragged by outliers, or outliers might get their own cluster instead of being ignored. Another limitation is that cluster analysis is simply a statistical technique – it assumes no underlying knowledge of the spatial structure. In other words, it is just clustering the data around a series of central points – which way it may or may not make sense once the analysis has been undertaken. The most important part of using the technique is the interpretation of the output to determine suitable strategies and measures to address underling issues by the planning side.

## 5 Conclusions

Our study showed distinct spatial disparities in heat exposure, green availability, and social status between the city districts of the research area. Less green districts are often inhabited by socially weaker populations and are more threatened by heat. Thus, our correlation analyses yielded strong and significant negative correlations between UHII and NDVI as well as between NDVI and the chosen social indicator (nonGerman). Heat and nonGerman, however, are characterized by a diffuse, relationship, varying from city to city (some coefficients indicating weak non-significant and some indicating strong significant positive correlations). Here, detailed studies and the inclusion and testing of further factors might enhance the vulnerability assessment for heat stress. The cluster analysis furthermore generated six distinguishable and spatially explainable clusters with similar characteristics regarding the researched components. We could show that in the study area, more people live in hot and less green districts than in cooler and greener ones. According to this, we can discern differently pronounced climate injustices in the researched cities of the Ruhr area, which will have to be addressed by the administration and planning side in the future.

Our methodical approach is characterized by its high portability and ease of use. Depending on research interest and data availability, other indicators can be included to potentially refine the analyses. Moreover, small-scale studies are needed, e.g., at the block level, as well as the consideration of other factors, such as the building structure or urban morphology in general. In addition, monitoring the described relationships is essential to be up-to-date and to notice changes. The described approach is suitable for presenting inter and intra-urban inequalities and issues in a generally understandable way. The relevance of vulnerability, multiple burdens, and inequality within the city and the region becomes visible, and combined with the local context, stimulates the necessary discussions on justice and the corresponding demands for action. Awareness for the shown spatial patterns and interactions is crucial for customized future urban planning and climate adaptation. On a broad level, we need efficient and unbiased approaches, standardized evaluation tools, and fundamentally accepted orientation values. The integration in planning tools such as heat action plans addressing climate injustice is essential. Therefore, for example, deficit and potential maps, designed also from a social perspective, are required. As a scientific analysis for climate policy decisions and planning in the management of climate adaptation, our work can support addressing climate injustice at an early planning stage. The study at hand is a contribution to the continuous development of procedures and methods in the field of climate adaptation planning for resilient, just and, healthy cities.

## Author contribution statement

Florian Klopfer; Antonia Pfeiffer: Conceived and designed the experiments; Performed the experiments; Analyzed and interpreted the data; Contributed reagents, materials, analysis tools or data; Wrote the paper.

## Data availability statement

The authors do not have permission to share data.

## Additional information

Supplementary content related to this article has been published online at [URL].

## Declaration of competing interest

The authors declare that they have no known competing financial interests or personal relationships that could have appeared to influence the work reported in this paper.
